# Supplementary material for: Historical and current distribution ranges and loss of mega-herbivores and carnivores of Asia
Source: PeerJ. 2021 Feb 16;9:e10738. doi: 10.7717/peerj.10738 (PMC7894109; doi:10.7717/peerj.10738)
Supplement: Supplemental Information 1 [file peerj-09-10738-s001.doc]

| **Section/topic** | **#** | **Checklist item** | **Reported on page #** |
| --- | --- | --- | --- |
| **TITLE** | | | 01 |
| Title | 1 | meta-analysis | 01 |
| **ABSTRACT** | | | 02 |
| Structured summary | 2 | Provide a structured summary including, as applicable: background; objectives; data sources; study eligibility criteria, participants, and interventions; study appraisal and synthesis methods; results; limitations; conclusions and implications of key findings; systematic review registration number.  **Structured Summary**  **Background:** Ecosystem functioning is dependent a lot on large mammals, which are, however, vulnerable and facing extinction risks due to human impacts mainly. Mega-fauna of Asia has been declining for a long, not only in numbers but also in their distribution ranges.  **Objectives**: In the current study, we aimed collecting information on past and current occurrence and distribution records of Asia’s megafauna species, to quantify mega-defaunation in Asia.  **Data sources**: Data sources included all published records available related to ten selected large mammal species. We also used the network of Protected Areas (PAs) in the region as a proxy for ‘healthy’ ecosystems where the intactness of faunal community and ecological processes should be desirable to quantify spatially explicit levels of mega-defaunation.  **Study eligibility criteria:** The published occurrence records of the selected large mammals of Asia were searched and collected. We also made sure that the records were authentic. This was done by looking at the authencity of the source reporting the species location such as peer reviewed journal, book information, newspaper information, personal communication etc. The articles or records based on weak evidence and location data were excluded from analysis in order to remove bias**.**  **Participants, and interventions:** We collected data on historical and current occurrences of ten selected large mammals of Asia using authentic published records, available on the internet, books, journals and so on.  **Study appraisal and synthesis methods:** After retrieving occurrence records of selected large mammals of Asia, the locations were fed in Google Earth to obtain the latitude-longitude geographic coordinates in the form of KML (Keyhole Markup Language) files, which were later exported to Quantum Geographic Information Systems (QGIS; Quantum GIS Development Team, 2012) and converted into shape files. The result was a layer of points with known historical presence of each species analyzed in the form of a map. Then, we used QGIS’ toggle editor tool to fill up gaps within the resulting ranges based on previously existing historical maps and ecological factors. Using these, we reconstructed the historical distribution ranges of the six herbivores and four carnivores for comparison with their present ranges. The current distribution of nine of the target species (all but Asiatic lions) was retrieved from IUCN’s Red List of Threatened Species website (http://www.iucnredlist.org/technical-documents/spatial-data) as a shape file document.  **Results:** The study revealed that the selected megafauna species were more widely distributed historically than at current. Severe range contraction was observed for Asiatic lion, three rhino species, Asian elephants, tigers, and tapirs. Defaunation maps generated have revealed vanishing of megafauna species from parts of the East. Southeast, and Southwest Asia, even the protected Areas losing up to eight out of ten large mammalian species.  **Limitations:** There were a couple of issues that could not be properly addressed in this study. First, we could not include as many species in our study as we have liked. Ideally, we would like to have included more wild bovids (e.g. banteng), large cats (e.g. snow leopard, Uncia uncia), bears, and large primates but were constrained by the availability of historical distribution data. We hope these gaps will be filled up in future studies. Secondly, the reconstruction of historical ranges was based on data obtained from different sources and the amount and quality of data available was highly variable across species, ranging from 458 historical distribution points for elephants to 29 in the case of tapirs. It is likely that our historical ranges differ from the real ones and they are more accurate for some species than for others, and for some areas than for others (e.g. depending on the availability of fossil records). Moreover, we focus on the loss of megafauna in PAs because much of the non-protected land in tropical Asia has been severely modified and occupied by humans, making it unsuitable for the presence of very large, often conflict-prone species, such as elephants and tigers. This is not to say that non-protected areas cannot or should not host megafauna, but it is more difficult to discriminate between areas that are suitable for megafauna and areas that are not. Finally, we also found difficulty in finding an appropriate metric to quantify defaunation since we used just changes in species richness without consideration of the particular species lost.  **Conclusions:** The selected large mammals of Asia were more widely distributed in the past and species including Asiatic lion, rhinos, elephants, tigers and tapirs have suffered severe contraction in their distribution ranges, even PA’s have suffered substantial mega-defaunation. The defaunation maps generated can be helpful developing future conservation policies, to save the remaining distribution ranges of large mammals. | 02 |
| **INTRODUCTION** | | | 03 |
| Rationale | 3 | Describe the rationale for the review in the context of what is already known.  Understanding and quantification of historic ranges of threatened megafauna is a prerequisite for the development of conservation and restoration policies (Laliberte & Ripple, 2004). Laliberte and Ripple (2004) assessed changes in the distribution range of 43 North American carnivores and ungulates since the 19th century and reported a loss of species richness and range contraction of > 20% in about one-third of the species. Ceballos and Ehrlich (2002) reported that among 173 threatened mammals from six different continents, have lost greater than 50% of their distribution ranges during the past two centuries.Globally, it is estimated that < 21% of the earth’s terrestrial surface has an intact assemblage of large mammals(> 20 kg) (Morrison et al., 2007). The Indomalayan region, having a great diversity of large mammals (Soberón & Ceballos, 2011; Ripple et al., 2016) has faced mammal decline (Ceballos & Ehrlich, 2002; Sodhi et al., 2010; Ripple et al., 2017) and has only maintained of intact large-mammal assemblage 1% on its terrestrial area (Morrison et al., 2007). Earlier studies have documented range contractions over time ranging from decades (Worm & Tittensor, 2011) to a few centuries (Laliberte & Ripple, 2004; Ceballos & Ehrlich, 2006; Morrison et al., 2007). The decline in Megafauna has been taking place in parts of tropical Asia, for several millennia (Elvin, 2004). The purpose of this study was to record the historical distribution of Asia’s megafauna over a period of approximately 10,000 years to identify the level of ‘mega-defaunation’ across the region and identify priority areas for conservation action. | 4 |
| Objectives | 4 | Provide an explicit statement of questions being addressed with reference to participants, interventions, comparisons, outcomes, and study design (PICOS).  Specifically, our objectives were to (1) collect data on the historical distribution ranges of selected Asian megafaunal species; (2) compare their historical and current distribution ranges; and (3) quantify megafaunal species loss in natural habitats, represented here by the network of Protected Areas (PAs) in the region. Our study provides spatially explicit information on ‘mega-defaunation’ levels that can be used in the design of conservation policies, particularly for the restoration of megafaunal populations and their ecological function. | 5 |
| **METHODS** | | | 6 |
| Protocol and registration | 5 | Indicate if a review protocol exists, if and where it can be accessed (e.g., Web address), and, if available, provide registration information including registration number.  N/A |  |
| Eligibility criteria | 6 | Specify study characteristics (e.g., PICOS, length of follow-up) and report characteristics (e.g., years considered, language, publication status) used as criteria for eligibility, giving rationale.  The geographical scope of our analyses was ‘Asia’ in *senso lato*. Specifically, we considered mainland Asia up to approximately 35° west and 40° north (we are aware that this is further north than standard tropical limits; e.g. Corlett (2013) and the islands of Sri Lanka, Sumatra, Borneo, Java, Hainan, and Taiwan (Fig. 1a). We consider ‘historical distribution’ as the natural occurrence of a species anytime in the past ~10,000 years. | 5 |
| Information sources | 7 | Describe all information sources (e.g., databases with dates of coverage, contact with study authors to identify additional studies) in the search and date last searched.  The location data on historical distribution/occurrence of the ten target megafaunal species were collected from published and unpublished literature, our sources included journal articles, books, research thesis, newspaper articles, and personal communications with reputable scientists. | 6 |
| Search | 8 | Present full electronic search strategy for at least one database, including any limits used, such that it could be repeated.  The Asian elephant (*Elephas maximus*) distribution, The Asian elephant (*Elephas maximus*) ecology, The Asian elephant (*Elephas maximus*) population, location, conflict, perception  Indian or greater one-horned rhinoceros (*Rhinoceros unicornis*), distribution, ecology, population, threats, location, conflict, perception  Javan or lesser one-horned rhinoceros (*Rhinoceros sondaicus*) distribution, ecology, population, threats, location, conflict, perception  Sumatran or two-horned Asian rhinoceros (*Dicerorhinus sumatrensis*) distribution, ecology, population, threats, location, conflict, perception  Gaur (*Bos gaurus*) distribution, ecology, population, threats, location, conflict, perception  Malayan tapir (*Tapirus indicus*) distribution, ecology, population, threats, location, conflict, perception  Tiger (*Panthera tigris*) distribution, ecology, population, threats, location, conflict, perception  Asiatic lion (*Panthera leo persica*) distribution, ecology, population, threats, location, conflict, perception  Common leopard (*Panthera pardus*) distribution, ecology, population, threats, location, conflict, perception  Clouded leopard (*Neofelis nebulosa)* distribution, ecology, population, threats, location, conflict, perception | 5-6 |
| Study selection | 9 | State the process for selecting studies (i.e., screening, eligibility, included in systematic review, and, if applicable, included in the meta-analysis).  The articles or records having weak evidence and location data were excluded from the analysis to remove bias and only those sources and records were considered having accurate location data of target species (Mahmood et al., 2019). | 6 |
| Data collection process | 10 | Describe method of data extraction from reports (e.g., piloted forms, independently, in duplicate) and any processes for obtaining and confirming data from investigators.  We collected historic location data on distribution of focal species were collected as described by Mahmood et al., (2019). Data on ecological parameters were also collected including vegetation type, altitude, etc., when available. | 6 |
| Data items | 11 | List and define all variables for which data were sought (e.g., PICOS, funding sources) and any assumptions and simplifications made.  We collected past occurrence (location) data for this study | 6 |
| Risk of bias in individual studies | 12 | Describe methods used for assessing risk of bias of individual studies (including specification of whether this was done at the study or outcome level), and how this information is to be used in any data synthesis.  Initially, we downloaded 2832 occurrence records of megafauna in different forms. After removing duplicate records, we were left with 2450 documents which were further screened and 903 documents were excluded based on weak evidence, and incomplete information. The remaining 1547 articles were further assessed, and 237 further articles were removed based on weak evidence and location data to remove bias and remaining 1310 articles which were used for quantitative synthesis and meta-analysis (Figure 1).  The articles or records having weak evidence and location data were excluded from the analysis to remove bias and only those sources and records were considered having accurate location data of target species (Mahmood et al., 2019). | 6 |
| Summary measures | 13 | State the principal summary measures (e.g., risk ratio, difference in means).  Not applicable | n/a |
| Synthesis of results | 14 | Describe the methods of handling data and combining results of studies, if done, including measures of consistency (e.g., I^2^) for each meta-analysis.  All data were collected and arranged in excel sheets for each species | 6 |

Page 1 of 2

| **Section/topic** | **#** | **Checklist item** | **Reported on page #** |
| --- | --- | --- | --- |
| Risk of bias across studies | 15 | Specify any assessment of risk of bias that may affect the cumulative evidence (e.g., publication bias, selective reporting within studies).  The articles or records having weak evidence and location data were excluded from the analysis to remove bias and only those sources and records were considered having accurate location data of target species (Mahmood et al., 2019). | 6 |
| Additional analyses | 16 | Describe methods of additional analyses (e.g., sensitivity or subgroup analyses, meta-regression), if done, indicating which were pre-specified.  We just mapped current and historic occurrences of species of interest and all analysis is given in methods | 5-8 |
| **RESULTS** | | | 8 |
| Study selection | 17 | Give numbers of studies screened, assessed for eligibility, and included in the review, with reasons for exclusions at each stage, ideally with a flow diagram.  Initially, we downloaded 2832 occurrence records of megafauna in different forms. After removing duplicate records, we were left with 2450 documents which were further screened and 903 documents were excluded based on weak evidence, and incomplete information. The remaining 1547 articles were further assessed and 237 further articles were removed based on weak evidence and location data to remove bias and remaining 1310 articles which were used for quantitative synthesis and meta-analysis (Figure 1). | 8 |
| Study characteristics | 18 | For each study, present characteristics for which data were extracted (e.g., study size, PICOS, follow-up period) and provide the citations.  The location data on historical distribution/occurrence of the ten target megafaunal species were collected from published and unpublished literature, our sources included journal articles, books, research thesis, newspaper articles, and personal communications with reputable scientists. Historic location data on the distribution of focal species were collected as described by Mahmood et al., (2019). Data on ecological parameters were also collected including vegetation type, altitude, etc., when available. The articles or records having weak evidence and location data were excluded from the analysis to remove bias and only those sources and records were considered having accurate location data of target species (Mahmood et al., 2019). | 8-14 |
| Risk of bias within studies | 19 | Give numbers of studies screened, assessed for eligibility, and included in the review, with reasons for exclusions at each stage, ideally with a flow diagram.  Initially, we downloaded 2832 occurrence records of megafauna in different forms. After removing duplicate records, we were left with 2450 documents which were further screened and 903 documents were excluded based on weak evidence, and incomplete information. The remaining 1547 articles were further assessed and 237 further articles were removed based on weak evidence and location data to remove bias and remaining 1310 articles which were used for quantitative synthesis and meta-analysis | 8 |
| Results of individual studies | 20 | For all outcomes considered (benefits or harms), present, for each study: (a) simple summary data for each intervention group (b) effect estimates and confidence intervals, ideally with a forest plot.  N/A |  |
| Synthesis of results | 21 | Present results of each meta-analysis done, including confidence intervals and measures of consistency.  N/A |  |
| Risk of bias across studies | 22 | Present results of any assessment of risk of bias across studies (see Item 15).  The articles or records having weak evidence and location data were excluded from the analysis to remove bias and only those sources and records were considered having accurate location data of target species. | 8 |
| Additional analysis | 23 | Give results of additional analyses, if done (e.g., sensitivity or subgroup analyses, meta-regression [see Item 16]).  N/A |  |
| **DISCUSSION** | | | 14 |
| Summary of evidence | 24 | Summarize the main findings including the strength of evidence for each main outcome; consider their relevance to key groups (e.g., healthcare providers, users, and policy makers).  This is, to our knowledge, the first attempt to produce a spatially explicit description of megafaunal loss in tropical Asia in historical times. We found that seven of the ten species in our analyses have suffered drastic range reduction in historical times. These are shocking figures that show the dire situation of Asian megafauna and the tendency towards a *neotropicalization* (a term coined by Richard Corlett) of tropical Asia. Importantly, we show that megafaunal loss has occurred not only in human-dominated landscapes but also in PAs – areas explicitly devoted to the conservation of biodiversity and ecological processes. Our results show a regional-scale case of megafaunal-empty forest (Redford, 1992) and a caveat of the current system of PAs in protecting ecological processes and interactions.  Larger species – whether herbivores or carnivores – had larger original distribution ranges and have also suffered the most acute range reductions. This contrasts with the results of Ceballos & Ehrlich (2002), who found no effect of body size in the range contraction patterns of 173 mammal species across the globe. Among megaherbivores, the three rhinoceros species have suffered the most dramatic range reductions, indicating that they are an especially vulnerable clade. Rhinos have been long persecuted in Asia for the medicinal value falsely attributed to their horns (Ellis, 2006). At present, rhinos can be considered ecologically extinct sensu (McConkey & Drake, 2006) throughout most Asian ecosystems and chances are that within the next few decades Sumatran and Javan rhinos will become extinct, both in the wild and captivity. Such a tragedy would be in line with the trend in the past few decades in which several rhino taxa have been declared extinct in the wild: mainland Javan rhinos in 2010, northern white rhinos in 2010 (*Ceratotherium cottoni*), (Emslie, 2012), and western black rhinos *Diceros bicornis longipes* in 2011 (Emslie, 2012). All these taxa were driven to extinction by human persecution. Although an alternative view suggests that rhinos got disappeared from their historical range in China due to mainly climatic factors (Elvin, 2004), we do not think that climate has played an important role compared with hunting and direct human competition for good habitats during the study period.  Asian elephants, the largest of Asian terrestrial animals, have shown dramatic range contraction which according to a previous estimate is > 95% by Sukumar (2006). Most of this loss occurred in southwest Asia (Turkey, Iraq, Iran, Afghanistan, and Pakistan), where elephants disappeared a long time ago (Olivier, 1978) as well as in China, where elephants have been gradually ‘retreating’ over the past 2.5 – 3 thousand years until remaining isolated in a small area of Yunnan’s province (Olivier, 1978; Elvin, 2004). Elephants got extinct from Java in the 18^th^ century (Cranbrook, Payne & Leh, 2008). In India, where approximately 60% of the remaining wild Asian elephant population occurs nowadays (Sukumar, 2006), they have also lost most of the range. In other parts of tropical Asia, the elephant range has become highly fragmented in recent times, e.g. in Sumatra they have recently been declared critically endangered after losing nearly two thirds of the subspecies habitat in one elephant generation (Gopala et al., 2011). Bornean elephants are considered native now.  Asian tapirs are one of the few Asian megafaunal species that are not persecuted for Chinese Traditional Medicine (Kawanishi & Sunquist, Melvin; Othman, 2002), and whose meat is not popular (especially in Malaysia, where they are considered non-halal, i.e. not permissible food under the Islamic law). For these reasons, there is a general assumption that tapir populations are not under high pressure (Kawanishi & Sunquist, Melvin; Othman, 2002). Our results, however, reveal a worrying situation with a dramatic reduction of 98% of their historical range and the complete disappearance from China, Laos PDR, Vietnam, Cambodia, and most of Myanmar and Thailand. Available data suggest that tapirs occur at relatively low densities, at least in Peninsular Malaysia (Rayan et al., 2012). Altogether, this depicts a more negative picture for tapir populations than often assumed.  Gaurs show the smallest range contraction among our studied megaherbivores, but this still amounts to almost three-quarters of their original range. Gaurs have probably been intensively hunted for their meat (Choudhury, 2002) throughout most of their range, to the point of being extirpated from Nepal, Bhutan, northern India, Bangladesh, sough China, and much of Indochina and the Malay Peninsula (Fig. 2b). Although the gaur was the only wild bovid included in this study, tropical Asia is home to other large and threatened wild bovids, notably the banteng (*Bos javanicus*; Endangered), kouprey (*Bos sauveii*; Critically Endangered and probably extinct), lowland anoa (*Bubalus depressicornis*; Endangered), mountain anoa (*Bubalus quarlesi*; Endangered), and the tamaraw (*Bubalus mindorensis*; Critically Endangered), among others. Most of these species have extremely reduced distribution ranges, often limited to island relic populations. We did not include these species in our analysis due to the difficulty to find information about their historical range.  A decline in the density of terrestrial herbivores in turn may threaten the largest carnivores like tigers, and the eventual loss of apex predators (trophic downgrading) leads to impacts that may cascade down through the food web. Among the four large carnivores studied, Asiatic lions lost almost all of their historical range and are now restricted to a single location in the Gir forest of India. Tigers have also got their ranges drastically reduced in history. In the last century alone, three tiger subspecies have been lost: the Caspian (*P. t. virgata*), Javan (*P. t. sondaica*), and Bali (*P. t. balica*) tigers, while the South China tiger (*P. t. amoyensis*) is probably extinct in the wild. Most of the range loss for tiger occurred in southwest Asia, Central Asia, and China. Sanderson et al. (2006) and Walston et al. (2010) have estimated that tigers lost 93% of their range, a figure very similar to our estimate in the current study. Much of this decline occurred in the last two centuries as the result of active persecution by colonial rulers. In French Indochina, for example, as many as 45,000 tigers could have been killed between 1860 and 1940 (Guérin, 2010). As many as 8,000 people might have been killed by tigers in Indochina during that same period (M. Guerin pers. comm.).  There seems to be a strong gradient of a higher diversity of megafaunal species in mainland East and Southeast Asia that declines towards the west (Fig. 3a). Historically, in some areas of Southeast Asia such as Taman Negara (Peninsular Malaysia) and Tonle Sap Biosphere Reserve (Cambodia) more than six of these megafaunal species occurred. The Himalayan Hills and the islands of Borneo and Sumatra are also areas with particularly high levels of megafaunal presence in historical times. The loss of megafauna has been most severe in parts of Indochina, East Asia, and the Himalayan Hills, where often more than five species of megafauna are missing in the Protected Areas (Fig. 4c).  We used Tropical Asia’s network of protected areas as a proxy for healthy – or at least conservation-relevant — ecosystems. We found that more than 90% of tropical Asia’s PAs have lost one or more megafauna species. These results coincide with previous studies that point out to tropical Asia, at least Southeast Asia, as a particularly sensitive area in terms of current defaunation patterns (Ceballos & Ehrlich, 2002; Morrison et al., 2007; Ripple et al., 2016, 2017). The results of our current study on mega defaunation can be compared with those that have shown that some areas of the world still retain intact mammal assemblages. For example, Morrison et al. (2007) compared the historical range maps of large mammals with their current distribution to determine areas that have retained complete assemblages of large mammals. They have shown that some regions of the world have been successful in keeping their fauna intact, 21% of terrestrial surface all of the large mammals more than 20 kg body weight once they contained. They also showed that 12% of the total area retaining large mammal assemblages are formerly protected, the degree of protection ranging from 9% in the Palearctic to 44% in the Indo-Malayan region. However, a key question regarding the loss of megafauna from Protected Areas is whether these species have been lost in these PAs before or subsequently to the establishment of the PAs. As it is evident from the history of Protected Areas established, since Yellow Stone National Park in USA, all Protected areas have brief history (few hundred years at maximum) of establishment, therefore, we cannot establish that megafaunal loss occurred from the protected areas, because after these areas were set out as protected, much more protection was available to the megafaunal species. Therefore, it is evident that megafaunal loss from PA’s had already occurred before these areas were set out as Protected Areas.  The large reductions in local megafaunal assemblages must have significant consequences for ecosystems. For example, Corlett (2013) showed co-extinctions of parasites and co-extinctions of commensalists and mutualists because host-specific commensalists and mutualists are also vulnerable. Similarly, Campos- Arceiz and Blake (2011) showed that both African (*Loxodonta* spp.) and Asian elephants have unique roles as long-distance dispersal agents for seeds of all sizes, including those too large for alternate frugivores to swallow. The next largest non-ruminant mammal in much of Southeast Asia, the Asian tapir, is unlikely to disperse large seeds from large fruits (Campos-Arceiz et al., 2012).  The loss of megaherbivores releases some plant resources for surviving competitors but feeding by megaherbivores may sometimes facilitate feeding by smaller species by increased browse availability near the ground (Makhabu, Skarpe & Hytteborn, 2006). The competitive interactions between predators can be complex and unpredictable but it has been documented that loss of top carnivores as apex predators results in “trophic downgrading (Estes et al., 2011). Megafaunal loss can also affect climate. For example, all mammalian herbivores produce methane (Franz et al., 2011) and that late Pleistocene spike in megafaunal declines resulted in a rapid loss in methane production, consequently triggering the abrupt younger dryas (12,800 – 11,500 B.P.) cooling event (Smith et al., 2010; Smith, Elliott & Lyons, 2011). However, carbon dioxide appears to be the primary driver of temperature changes at the end of last glacial period (Shakun et al., 2012). | 15-20 |
| Limitations | 25 | Discuss limitations at study and outcome level (e.g., risk of bias), and at review-level (e.g., incomplete retrieval of identified research, reporting bias).  Several issues could not be properly addressed in this study. First, we could not include as many species in our study as we have liked. Ideally, we would like to have included more wild bovids (e.g. banteng), large cats (e.g. snow leopard, *Uncia uncia*), bears, and large primates but were constrained by the availability of historical distribution data. We hope these gaps will be filled up in future studies. Second, the reconstruction of historical ranges was based on data obtained from different sources and the amount and quality of data available were highly variable across species, ranging from 458 historical distribution points for elephants to 29 in the case of tapirs. Our historical ranges likely differ from the real ones and they are more accurate for some species than for others, and for some areas than for others (e.g. depending on the availability of fossil records). Thirdly, we had difficulty in assessing the reliability of some of our historical records, as well as in assigning geographical locations to some records that were expressed loosely. Moreover, we focus on the loss of megafauna in PAs because much of the non-protected land in tropical Asia has been severely modified and occupied by humans, making it not suitable for the presence of very large, often conflict-prone species, such as elephants and tigers. This is not to say that non-protected areas cannot or should not host megafauna, but it is more difficult to discriminate between areas that are suitable for megafauna and areas that are not. Finally, we also found difficulty in finding an appropriate metric to quantify defaunation since we used just changes in species richness without consideration of the particular species lost. Recent work in objectively quantifying defaunation (Giacomini & Galetti, 2013) is very promising and we expect more work developing in this direction.  How robust are the earlier distribution records compiled here? Some other studies published have reported some biases in this regard, for example, Monsarrat et al (2018) demonstrated spatial biases in reporting of historical distributions of large mammals. Also, Monsarrat & Kerley (2018) also reported taxonomic biases in the historical reporting of large mammals. Therefore, in the current study, we do understand and realize and recognize the risks inherent in such biases and obviously, these may influence the study outcomes. This is especially the case given the absolute paucity of data for some of the species focused in the current study and across such a huge area, as well as the varying socio-political histories (and hence reporting prospects) across their study area. | 20-21 |
| Conclusions | 26 | Provide a general interpretation of the results in the context of other evidence, and implications for future research.  Our study provides an insight on defaunation/ range contraction of important herbivore and carnivore species and our findings can be used to guide conservation policies, especially for ecological restoration projects. Historically, the selected megafauna species were found more widely distributed than at current. By groups, rhinos showed the most dramatic range changes, followed closely by Asiatic lions, tapirs, tigers, and elephants. Defaunation was extreme in parts of East and Southeast Asia with Protected Areas having lost up to eight megafaunal species. | 22 |
| **FUNDING** | | |  |
| Funding | 27 | Describe sources of funding for the systematic review and other support (e.g., supply of data); role of funders for the systematic review.  No funding was available for this research work. | 22 |

*From:*  Moher D, Liberati A, Tetzlaff J, Altman DG, The PRISMA Group (2009). Preferred Reporting Items for Systematic Reviews and Meta-Analyses: The PRISMA Statement. PLoS Med 6(7): e1000097. doi:10.1371/journal.pmed1000097

For more information, visit: **www.prisma-statement.org**.

Page 2 of 2
