# Supplementary material for: Historical and current distribution ranges and loss of mega-herbivores and carnivores of Asia
Source: PeerJ. 2021 Feb 16;9:e10738. doi: 10.7717/peerj.10738 (PMC7894109; doi:10.7717/peerj.10738)
Supplement: Supplemental Information 2 [file peerj-09-10738-s002.doc]

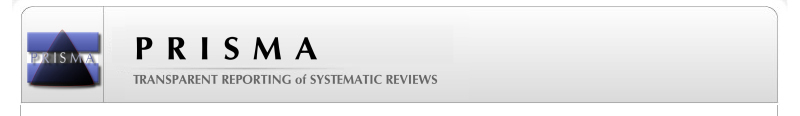
**PRISMA 2009 Flow Diagram**

**Screening**

**Included**

**Eligibility**

**Identification**

Records identified through database searching
(n = 2024 )

Additional records identified through other sources
(n = 808 )

Records after duplicates removed
(n = 2450)

Records screened
(n = 2450 )

Records excluded
(n = 903 )

Full-text articles assessed for eligibility
(n = 1547)

Full-text articles excluded, (Due to weak evidence)
(n =237 )

Studies included in qualitative synthesis
(n = 1310)

Studies included in quantitative synthesis (meta-analysis)
(n = 1310)
